# In Situ Ruthenium Catalyst Modification for the Conversion of Furfural to 1,2-Pentanediol

**DOI:** 10.3390/nano12030328

**Published:** 2022-01-20

**Authors:** Lauriane Bruna, Miquel Cardona-Farreny, Vincent Colliere, Karine Philippot, M. Rosa Axet

**Affiliations:** CNRS, LCC (Laboratoire de Chimie de Coordination), Université de Toulouse, UPS, INPT, 205 Route de Narbonne, CEDEX 04, 31077 Toulouse, France; bruna.lauriane98@gmail.com (L.B.); miquel.cardona@lcc-toulouse.fr (M.C.-F.); vincent.colliere@lcc-toulouse.fr (V.C.); karine.philippot@lcc-toulouse.fr (K.P.)

**Keywords:** nanocatalysis, ruthenium, furfural, biomass, pentanediol

## Abstract

Exploiting biomass to synthesise compounds that may replace fossil-based ones is of high interest in order to reduce dependence on non-renewable resources. 1,2-pentanediol and 1,5-pentanediol can be produced from furfural, furfuryl alcohol or tetrahydrofurfuryl alcohol following a metal catalysed hydrogenation/C-O cleavage procedure. Colloidal ruthenium nanoparticles stabilized with polyvinylpyrrolidone in situ modified with different organic compounds are able to produce 1,2-pentanediol directly from furfural in a 36% of selectivity at 125 °C under 20 bar of H_2_ pressure.

## 1. Introduction

**Scheme 1:**
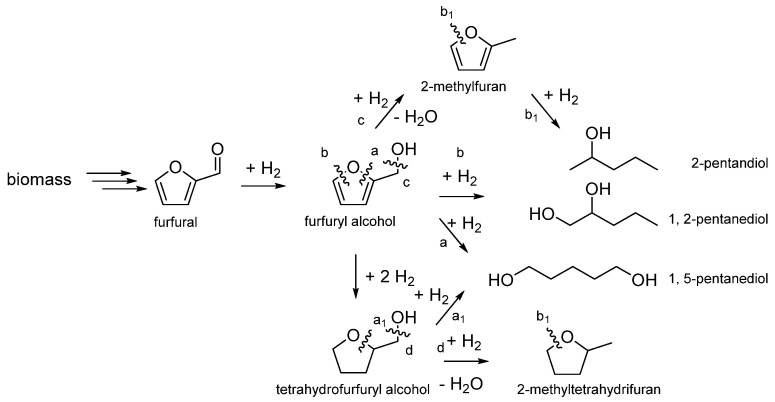
Simplified reaction scheme of the catalytic hydrogenation of biomass-derived furfural into several valuable products.

1,2-pentanediol (1,2-PeD) and 1,5-pentanediol (1,5-PeD) are interesting compounds due to their applications as monomers for polymer synthesis such as polyesters, polyurethanes, and polyamides, as well as potential fuels, or solvents [1]. Both can be produced from biomass-derived compounds, furfural (FR), or its hydrogenated derivatives furfuryl alcohol (FA) and tetrahydrofurfuryl alcohol (THFA), by hydrogenation followed by hydrogenolysis of a C-O bond (Scheme 1) [2,3,4,5]. This route has attracted attention as it can become an alternative to the use of oil-based products for their production [6,7]. Noble metal-based catalysts have been described to be highly efficient to produce these two diols from furfural or its hydrogenated derivatives, Pt- [8,9,10,11,12,13,14] and Ru-based [15,16,17,18,19] catalysts producing mainly 1,2-PeD, and Rh- and Ir-based [20,21,22] catalysts, 1,5-PeD. Cu-based [23,24,25] catalysts produce a mixture of both diols usually needing harsher reaction conditions. Pt-based catalysts efficiently cleavage the C-O bond of FA to produce 1,2-PeD, several of these catalysts producing this diol in high yields [8,9,10]. Already in 1949, platinum oxide was reported to produce quantitative amounts of 1,2-PeD in acetic acid from FA [8]. From a point of view of practicality, it is preferable to produce these two diols directly from furfural as it avoids a multistep procedure. In this case, Pt-based catalysts are still reasonably performant, leading to moderate to high yields of 1,2-PeD [11,12,13,14]. Recently, 1,5-PeD could be obtained using a Pt-based catalyst, Pt@Al_2_O_3_ in very mild reaction conditions, 45 °C in water, and NaBH_4_ as reducing agent [26]. The selectivity towards 1,5-PeD was of 75%, which is in contrast with the tendency of Pt-based catalysts to produce 1,2-PeD from FR or FA under H_2_. The different mechanism operating was investigated by the authors; NaBO_2_ and the Brønsted acidity of the support being key for the high selectivity towards 1,5-PeD. It is clear from the literature that the support of the heterogeneous Pt-catalysts plays an important role on the outcome of the reaction. As an illustration, Pt on ceria displayed better catalytic performances than Pt on silica, alumina or magnesia [14]. Size and shape of the Pt nanoparticles also have an effect on the selectivity in the hydrogenation of FA; monodisperse, 2D or 3D Pt catalysts afforded 2-methylfuran (2-MF), THFA, or 1,2-PeD, respectively [27]. Rh- and Ir-based catalysts have demonstrated exceptional efficiency for the production of 1,5-PeD from FR, FA or THFA. In 2009, Tomishigue’s group reported an outstanding catalyst, Rh-ReO*_x_*/SiO_2_, able to produced 1,5-PeD in very high yields from THFA [20]. Developing further these rhodium catalysts, this group demonstrated the possibility to obtain 1,5-PeD from furfural and the importance of the support or the oxide additive (Re, Mo or W) to control both activity and selectivity [21,22]. Cu-based catalysts are also effective for the production of pentanediols from FA and THFA, but in general requiring harsher reaction conditions than catalysts based on noble metals. For instance, a 10 wt % Cu-Mg_3_AlO_4.5_ afforded both diols in a 80% yield, 1,2-PeD in a 51% and 1,5-PeD in a 29% selectivity, at 99% conversion of FA at 140 °C under 60 bar of H_2_ [23]. Similarly, a 10 wt % Cu/Al_2_O_3_ catalyst gave a 86% conversion of FA with a 70% selectivity towards the pentanediols (48% 1,2-PeD and 22% 1,5-PeD) at 140 °C and 80 bar of H_2_ [24]. A series of CuMgA catalysts, whose properties were modulated by controlling the Mg content, were able to efficiently convert FR or FA at 140 °C and 60 bar of H_2_ to pentanediols [25]. Cu_1.8_Mg_1.2_Al converted 95% of FA into 46% of 1,2-PeD and 16% of 1,5-PeD, together with other minor by-products. Noticeable size effects or the effect of the number of basic sites on the activity were revealed, which in turn were controlled by the Mg content.

Ru-based catalysts have been studied less for the production of pentanediols from FR or its derivatives [15,16,17,18], even if this metal is able to perform hydrogenation reactions efficiently, at a reasonable price [28]. Zhang et al. [15] studied the aqueous-phase hydrogenolysis of FA to 1,2-PeD on a series of supported Ru catalysts (MnO_x_, MgO, MgAlO_4_, NaY, active carbon (AC), ZrO_2_ and TiO_2_), and Pd, Pt and Rh over MnO_x_. Ru/MnO_x_ displayed the best catalytic performances affording 1,2-PeD in a 42% yield at 150 °C and 15 bar of H_2_ in water. Besides the nature of the catalyst other reaction parameters were studied, allowing to propose a mechanism for the formation of 1,2-PeD, which would be produced from the partially hydrogenated product, dihydrofurfuryl alcohol (DHFA). Recently, it has also been suggested that DHFA is a key intermediate for the formation of 1,2-PeD when using a Pt supported on Mg(Al)O catalyst [27]. Yamaguchi et al. [19] performed a similar study with Ru-supported catalysts for the hydrogenolysis of FA reporting similar conclusions of those of the work of Zhang et al. [15]. Götz et al. [16] studied a series of Ru-supported catalysts (carbon, silica, and alumina) for the same reaction, but using harsher reaction conditions (200 °C, 100 bar of H_2_) compared to the work of Zhang et al. [15], in this case affording up to 32% yield of 1,2-PeD. It is worth noting that in aqueous media, significant amounts of polymerized product were produced, in contrast with the work of Zhang et al. [15] in which that side reaction was supressed by the use of a basic support. Ru-Mn/CNTs catalyst has also been reported as active for the hydrogenation/hydrogenolysis of FA, producing THFA and 1,2-PeD, the latter with a selectivity up to 20%. In a previous work carried out by some of us [29], Ru/PVP (polyvinylpyrrolidone) colloidal catalyst displayed a good efficiency to produce 1,2-PeD from FA. After 22 h of reaction at 125 °C under 20 bar of H_2_, FA in the presence of Ru/PVP in 1-propanol quantitatively produced THFA and 1,2-PeD in a 73% and 27%, respectively, without any by-product (Figure S1). Taking into consideration this previous result, we examine here the possibility of using Ru/PVP catalysts to directly transform furfural into 1,2-PeD. Knowing that this transformation is sensitive to support effects [9,14,15] and our experience of tuning activity and selectivity in catalysed reactions by changing the adsorbates on the metallic surfaces [30,31,32,33], we have studied the effect of the in situ addition of organic ligands in order to examine their impact in the outcome of this chemical transformation. The modulation of the catalytic properties of heterogeneous catalysts using surface adsorbates is an important field in catalysis, yet it is in its infancy [34]. The adsorbates are key parameters to modulate a plethora of structural and reactivity parameters of metal surfaces, and in this sense, it is sometimes difficult to drive exact conclusions on the phenomenon, especially the effect on catalysis. Even so, it has been demonstrated in some works that organic ligands are able to change the outcome of catalysed reactions when compared to bare metallic surfaces, or increasing activity or favouring the synthesis of a selected product [31,35,36,37]. Interesting enough, recently some works point out that basic ligands together with acidic metallic surfaces could form frustrated Lewis pairs (FLP), which will be responsible for the modulation of the reactivity of metallic surfaces [38,39,40]. This phenomenon, not yet fully studied, adds complexity to the understanding of the role of ligands in heterogeneous catalysis.

## 2. Experimental Section

### 2.1. General Methods

All operations were carried out under argon atmosphere using standard Schlenk techniques or in an MBraun glovebox. Solvents were purified by standard methods or by an MBraun SPS-800 solvent purification system. [Ru(η^4^-C_8_H_12_)(η^6^-C_8_H_10_)] was purchased from Nanomeps Toulouse, polyvinylpyrrolidone (PVP, mol wt 40,000), hexadecylamine (HDA), 2,2,6,6-tetramethylpiperidine (TMP), *tert*-butylamine (TBA), furfural, tetrahydrofuran (THF) and dodecane from Sigma-Aldrich, furfuryl alcohol from Alfa Aesar, diphenyl-2-pyridylphosphine from TCI, H_2_ from Air Liquid. All of these reactants were used as received. NHC was synthesised following the procedure described elsewhere [41]. Metal content was established by inductively coupled plasma optical emission spectroscopy (ICP-OES) performed at the “Laboratoire de Chimie de Coordination, Toulouse” in a Thermo Scientific ICAP 6300 instrument. Liquid NMR measurements were performed on a Bruker Avance 300 instrument or Bruker Avance 400 intrument. ATR-IR spectra were recorded on a Perkin-Elmer GX2000 spectrometer available in a glovebox, in the range 4000–400 cm^−^^1^. TEM analyses were performed at the “Centre de microcaracterisation Raimond Castaing, UMS 3623, Toulouse” by using a JEOL JEM 1011 electron microscope operating at 100 kV with a point resolution of 4.5 Å or a JEOL JEM 1400 operating at 120 kV with a point resolution of 2.0 Å. The approximation of the particles mean size was stablished through a manual analysis of enlarged micrographs by measuring at least 200 particles on a given grid of copper. Quantitative analyses of the catalytic reaction mixtures were performed via GC analyses using internal standard technique and solutions of commercially available products. GC analyses were performed on a SHIMADZU GC-2014 equipped with a SUPELCOWAX 10 capillary polar column (30 m × 0.25 mm × 0.25 μm). The method used for furfural reaction mixture analyses consists on: carrier gas flow, He, 1.25 ml/min; injector temperature, 250 °C; detector (FID) temperature, 250 °C; oven program, 50 °C (hold 3 min) to 240 °C at 20 °C/min (hold 10 min) for a total run time of 22.5 min; retention time, dodecane, 6.8 min; furfural, 9.2 min; tetrahydrofurfuryl alcohol, 9.4 min; acetal, 10.0 min; furfuryl alcohol, 10.4 min; 2-propoxymethyl furan, 10.6 min; and 1,2-pentanediol, 11.1 min. GC-MS analyses were performed in a Shimadzu QP2010 Ultra GC-MS (EI mode), equipped with a ZEBRON ZB-5ms capillary column (30 m × 0.25 mm × 0.25 μm). The method used for furfural reaction mixture analyses consists on: carrier gas flow, He, 1 mL/min; injector temperature, 250 °C; detector (FID) temperature, 250 °C; oven program, 40 °C (hold 0.5 min) to 250 °C at 20 °C/min (hold 10 min) for a total run time of 21 min; retention time: furfural, 3.6 min; furfuryl alcohol, 3.8 min; tetrahydrofurfuryl alcohol, 4.0 min; 1,2-pentanediol, 4.3 min; 2-propoxymethyl furan, 4.7 min; dodecane, 6.3 min; and acetal, 6.7 min. 

### 2.2. Synthesis of Ru/PVP

A total of 140 mg (0.445 mmol) of [Ru(η^4^-C_8_H_12_)(η^6^-C_8_H_10_)] complex was introduced in a Fisher-Porter bottle, together with 150 mg of PVP, and then dissolved in 40 mL of THF in a glovebox. The yellow solution reacted with 3 bar of H_2_ at room temperature. After some minutes, the solution turned black. The reaction was kept overnight under vigorous stirring. After this period of time, the H_2_ excess was removed and pentane was added to precipitate the NP. After filtration under argon with a cannula, the black solid powder was washed twice with pentane and filtered again before drying under reduced pressure overnight. Yield: 79 mg. ICP anal.: 20.08% Ru. 

### 2.3. Catalytic Hydrogenations

The hydrogenation of furfural was carried out in a 50 mL stainless steel high-pressure batch Top Industrie reactor. In a typical experiment, a mixture of the catalyst (0.02 mmol of metal), dodecane (0.5 mmol) as internal standard, and furfural (4 mmol) as substrate in 15 mL of 1-propanol were loaded into the autoclave in the glovebox. In catalysis carried out in the presence of a ligand, 0.06 mmol of HDA, TMP, or TBA, or 0.03 mmol of PN, or 0.02 mmol of NHC, were added to the dispersion. The autoclave was purged three times with H_2_ to remove the inert atmosphere, heated to the desired temperature, and charged with 20 bar of H_2_. Reactions carried out at 3 bar of H_2_ pressure were performed in a Fisher-Porter bottle, a mixture of the catalyst (0.013 mmol of metal), dodecane (0.33 mmol) as internal standard, and furfuryl alcohol (2.7 mmol) as substrate in 10 mL of 1-propanol were loaded into the bottle in the glovebox. The vessel was purged three times with H_2_ to remove the inert atmosphere, heated to the desired temperature, and charged with 3 bar of H_2._ The stirring rate was fixed at 1200 rpm. Samples of the reaction mixture were taken at different time intervals and analysed by gas chromatography. Quantitative analyses of the reaction mixtures were performed via gas chromatography using calibration solutions of commercially available products.

## 3. Results and Discussion

**Figure 1:**
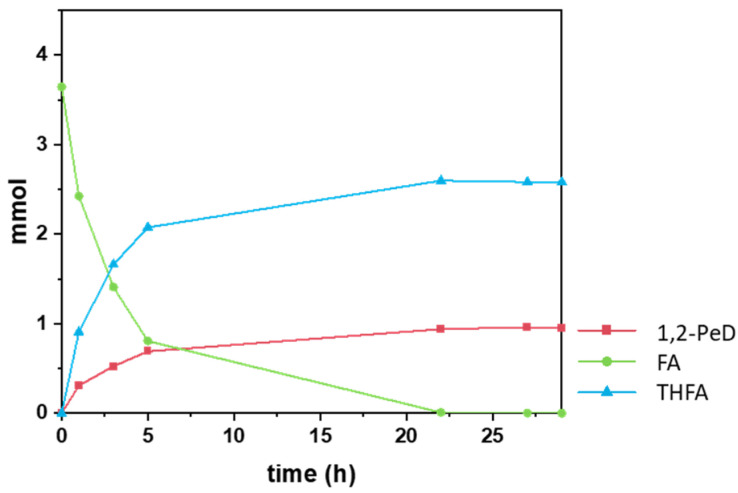
Time–concentration curve for furfuryl alcohol hydrogenation using Ru/PVP as catalyst. Furfural (red curve), furfuryl alcohol (green curve), tetrahydrofurfuryl alcohol (blue curve), acetal (orange curve), 1,2-pentanediol (pink curve). Reaction conditions: 2 × 10^−^^2^ mmol of Ru, 4 mmol of FA, 0.5 mmol of dodecane (internal standard), 20 bar of H_2_, 125 °C, 15 mL of 1-propanol. Quantities of products and reagents were determined by GC using an internal standard technique [29].

As stated above, Ru/PVP catalyst efficiently hydrogenates the C=O bond of FR in 1-propanol at 125 °C under 20 bar of H_2_ [29]. The heteroaromatic ring starts to be hydrogenated in turn after 5 h of reaction (Figure S1). This stepwise hydrogenation is rather selective, but the formation of considerable amounts of acetal by reaction of the aldehyde with the alcoholic solvent in the first stages of the reaction, make the overall selectivity low. If the FA hydrogenation is independently studied using the same reaction conditions, it is observed that 1,2-PeD is produced in a 27% selectivity together with THFA showing a TOF of 79 h^−1^ without the production of any by-product (Figure 1).

**Figure 2:**
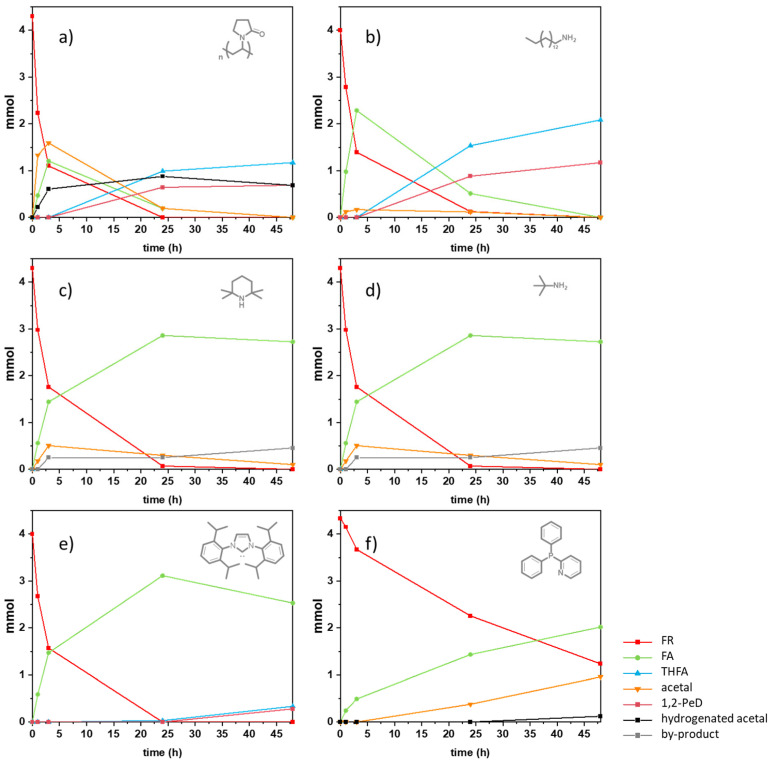
Time–concentration curves for furfural hydrogenation using in situ modified Ru/PVP as catalyst together with unmodified Ru/PVP. (**a**) Ru/PVP; (**b**) Ru/PVP modified with HDA; (**c**) Ru/PVP modified with TMP; (**d**) Ru/PVP modified with TBA; (**e**) Ru/PVP modified with NHC; and (**f**) Ru/PVP modified with PN. Furfural (red curve), furfuryl alcohol (green curve), tetrahydrofurfuryl alcohol (blue curve), acetal (orange curve), 1,2-pentanediol (pink curve), hydrogenated acetal (black curve), unidentified products (grey curve). Reaction conditions: 2 × 10^−^^2^ mmol of Ru, 4 mmol of FR, 0.5 mmol of dodecane (internal standard), (**b**) 6 × 10^−^^2^ mmol of HDA, (**c**) 6 × 10^−^^2^ mmol of TMP (**d**) 6 × 10^−^^2^ mmol of TBA, (**e**) 2 × 10^−^^2^ mmol of NHC, (**f**) 3 × 10^−^^2^ mmol of PN, 20 bar of H_2_, 125 °C, 15 mL of 1-propanol. Quantities of products and reagents were determined by GC using an internal standard technique.

In a previous work carried out by some of us, the selective hydrogenation of cinnamaldehyde was investigated by using an electron-deficient Ru nanocatalyst, Ru/C_60_ [42]. This Ru catalyst was prone to promote the acetalisation reaction of the cinnamaldehyde with the alcoholic solvent due to its electron deficiency. By adding a base to the reaction media, this reactivity was supressed, and the selectivity of the hydrogenation reaction of cinnamaldehyde was modulated depending on the base used, which was attributed to the increase in electronic density of the metallic surface by the presence of the base. With the aim to use FR as the starting compound to produce 1,2-PeD, and avoid thus, a multistep procedure, the reaction of the aldehyde with 1-propanol to give the corresponding acetal must be supressed to improve the selectivity (Figure S1). According to that, we decided to use several electron-donating ligands in order to increase the electron density on the Ru NP surface to hamper the acetalisation reaction, as bare Ru NPS, as Ru/PVP, only weakly stabilized by the polymer, are electron deficient due to the presence of hydrides on the surface [29,43,44]. For this purpose, a long chain amine, hexadecylamine (HDA), two bulky amines, 2,2,6,6-tetramethylpiperidine (TMP), and *tert*-butylamine (TBA), a N-heterocyclic carbene, 1,3-bis(2,6-diisopropylphenyl)imidazole-2-ylidiene (NHC), and a phosphine containing a pyridine moiety, diphenyl-2-pyridylphosphine (PN) have been chosen, all being electron donor ligands able to coordinate to the Ru NP surface [28,30]. In addition to the suppression of the acetal, the impact of ligands adsorbed in the metallic surface in this reaction was assessed. The investigation of the role of the ligands on heterogeneous catalysis is a crucial topic [30,34]. Nevertheless, as the ligands/stabilizers have an impact modulating the size, shape, chemical, and crystalline structure, among other parameters and properties of the metallic surfaces and nanoparticles [45], it is difficult to compare its exact role in catalysis. To minimise the impact of the ligands in structural parameters, they were added directly to the catalytic medium, in order to in situ modify the Ru/PVP nanocatalyst. The results on the selective hydrogenation of furfural towards 1,2-PeD using modified Ru/PVP are summarized in Table 1. Time–concentration curves of the reactions are presented in Figure 2.

Ru/PVP is an efficient catalyst for the hydrogenation of furfural, which its aldehyde moiety was hydrogenated to the corresponding alcohol with a TOF of 55 h^−1^. In 5 h, the aldehyde was almost completely consumed to give FA and the corresponding acetal (Figure 2a). After 24 h of reaction, FA is nearly all reacted producing mainly THFA and 1,2-PeD in 29% and 19% selectivity, respectively. Besides that, the heteroaromatic ring of the acetal was hydrogenated to produce the corresponding hydrogenated acetal (black line, Figure 2a). After 48 h of reaction, the selectivity towards 1,2-PeD slightly increased to 24%, probably due to the fully consumption of FA. THFA selectivity also increased to 42%, which it is tentatively attributed to the same reason and to the production of it from the hydrogenated acetal.

The addition of an amine ligand to the reaction media increased the TOF of the FR hydrogenation compared to the unmodified Ru/PVP catalyst. HDA, TMP, and TBA showed TOFs of 99 h^−1^, 71 h^−1^, and 66 h^−1^, respectively, HDA increasing the TOF substantially, almost twice the one without any surface modifier (Table 1, entry 5 vs. entry 1). We attribute that to the increase in electron density of the Ru surface by the coordination of the amines to the metallic surface [30,31,34,35,37]. It is interesting to take into consideration that amines weakly coordinate to the surface of the metallic nanoparticles as shown experimentally and theoretically in previous works [31,46], and for that reason are in equilibrium with the solution. Amines and phosphines have been described to form frustrated Lewis pairs (FLP) [40] with the acidic metallic surfaces of gold [38] and nickel-copper [39] for instance, and are able to activate H_2_ or Si–H bonds effectively into those surfaces. We cannot discard that this phenomenon is also occurring in our catalytic media as amines are in excess with respect to the ruthenium atoms, thus FLP interactions could help to accelerate the reaction. Nevertheless, the addition of three equivalents of amine with respect with Ru, drastically supressed the formation of acetal. This can be attributed to the increase in electronic density of the ruthenium surface due to the presence of a basic ligand. The addition of the NHC also supressed the formation of the acetal, having a beneficial effect on selectivity, as FA was quantitatively obtained (Table 1 entries 21–22). The activity remained similar to the unmodified Ru catalysts, as TOF at 1 h of reaction was of 51 h^−1^.

Using HDA as surface modifier allowed the hydrogenation reaction to proceed further, 85% of FA was consumed after 24 h of reaction to give rise to THFA in a 45% of selectivity and 1,2-PeD in a 26% of selectivity (Table 1 entry 7). After 48 h the reaction mixture remained almost identical, producing 64% of THFA together with 36% of 1,2-PeD. This result is very stimulating as it is very close to the best reported selectivity for 1,2-PeD using a Ru-based catalyst (42% using Ru/MnOx as catalyst [15]), but in our case directly from furfural.

In contrast to Ru/PVP and Ru/PVP modified with HDA, the addition of the TMP, TBA or NHC highly impeded the hydrogenation or opening of the heteroaromatic ring of FA and only 20% of THFA was produced after 24 h of reaction in the case of NHC, while for the bulky amines the reaction did not proceed. This behaviour was tentatively attributed to the high steric hindrance of these molecules; nevertheless, this hypothesis has to be further confirmed. The last ligand chosen, the phosphine containing a pyridine moiety (PN), largely affected the activity in the hydrogenation of the aldehyde moiety of FR, with a TOF of 15 h^−1^, which is in contrast with the other catalytic systems of this work, indicating a strong interaction of the ligand with the surface. This is in line with previous results by Chaudret et al. [47], which demonstrated using spectroscopic techniques that phosphines coordinated strongly to the Ru surface. The acetalisation reaction was also strongly influenced by the presence of the phosphine; nevertheless, not completely suppressed, as in the case of using HDA or NHC as surface modifier. After 48 h of reaction, the conversion of FR was only of 69%, with a selectivity of 58% of FA and 27% of acetal.

According to the results, the addition of a basic ligand supresses the formation of the acetal by reaction of FR with 1-propanol, which was the first aim of this work. In the case of NHC, only one equivalent is needed to avoid this side reaction, while for HDA, TMP, and TBA three equivalents were required. We have attributed that to the different basicity of the compounds as well as the robustness of the bond to the surface. It has been demonstrated that NHC coordinates strongly to the metallic surfaces [48], while HDA is in equilibrium with the solution [46]. It seems that both kinds of ligands are donor enough to increase the electron density of the metallic surface, and in consequence avoid the acetalisation reaction. On the other hand, the fact that NHC gives not as high TOF as HDA-modified Ru catalyst suggests that the robustness of the bond of the NHC towards the surface together with its high steric hindrance are detrimental for the coordination of the substrate. This could be also the scenario for the hydrogenation/C-O cleavage of the heteroaromatic ring, which proceeds sluggishly using bulky compounds to modify the Ru catalyst. PN-modified Ru catalyst was the least active of the series of catalysts and did not prevent the formation of the acetal, even if using the same ratio of donor atoms than in the case of amines. We have tentatively attributed this behaviour to its high steric on one hand, whilst the pyridine moiety, less basic that HDA, is not coordinating strongly enough to supress the formation of the acetal. HDA-modified Ru catalyst thus allows to produce FA efficiently and selectively, while promoting as well the C-O cleavage. Ru/PVP produces 27% and 24% of 1,2-PeD from FA and FR, respectively, while HDA-modified Ru/PVP provides up to 36%, which is a substantial increase.

In order to assess the stability of the catalysts, the size and morphology of the spent catalysts have been analysed by TEM and the Ru content of the filtrated after catalysis analysed by ICP. All data are summarized in Table S1 and TEM images (Figures S2–S8) after catalysis are given in the SI. The size of the catalysts remained essentially the same after the hydrogenation reaction. The ICP analysis displayed the presence of Ru in the supernatant, but always under the quantification limit, making them not accurate enough for quantification. Nevertheless, an estimation of the leached catalyst was calculated and is given in the SI, showing a minor leaching; usually below 1% of Ru. Ru/PVP is very dispersed on 1-propanol and a recycling test could not be performed.

## 4. Conclusions

Ru/PVP modified in situ with an amine is a promising catalyst to produce 1,2-PeD directly from furfural. This is the first time that a Ru catalyst produces this diol directly from furfural in an interesting selectivity (36%). Even if the catalyst is not suitable yet for industrial applications, as it is difficult to recycle and is activity is overall low, we have shown that a straightforward modification, only the use of an amine, is an interesting way to modulate the selectivity and activity of the system. We have shown as well that the in situ modification of heterogeneous catalysts is possible and the high impact that it produces in the outcome of this hydrogenation reaction, allowing a straightforward comparison as the initial catalyst is the same in all cases.

## Figures and Tables

**Table 1:** Selective hydrogenation of furfural in 1-PrOH using Ru/PVP nanocatalysts in situ modified. ^a^

Entry	Catalyst	TOF (h^−1^) ^b,c^	Time (h)	Conversion (%) ^c^	FA (%)	Acetal (%) ^c^	THFA (%) ^c^	1,2-PeD (%) ^c^
1	Ru/PVP	117 (55)	1	44	23	77	-	-
2			3	72	35	65	-	-
3			24	100	6	32	29	19 *
4			48	100	0	24	42	24 *
5	Ru/PVP + HDA	99	1	31	89	11	-	-
6			3	65	93	7	-	-
7			24	97	15	3	45	26 *
8			48	100	0	-	64	36
9	Ru/PVP + TMP	71	1	27	94	3 *	-	-
10			3	72	90	4 *	-	-
11			24	100	93	3	-	-
12			48	100	90 *	-	-	-
13	Ru/PVP + TBA	66	1	26	71	22 *	-	-
14			3	56	66	23 *	-	-
15			24	98	84	9 *	-	-
16			48	100	83	3 *		
17	Ru/PVP + PN	15	1	4	>99	-	-	-
18			3	15	>99	-	-	-
19			24	44	79	21	-	-
20			48	69	58	27	-	- *
21	Ru/PVP + NHC	51	1	20	>99	-	-	-
22			3	52	>99	-	-	-
23			24	100	99	-	>1	-
24			48	100	80	-	11	9

^a^ Reaction conditions: 2 × 10^−^^2^ mmol of metal, 4 mmol of furfural, 0.5 mmol of dodecane (internal standard), 20 bar of H_2_, 125 °C, 15 mL of 1-propanol; entries 5–8, 6 × 10^−^^2^ mmol of HDA, entries 9–12, 6 × 10^−^^2^ mmol of TMP, entries 13–16, 6 × 10^−^^2^ mmol of TBA, entries 17–20, 3 × 10^−^^2^ mmol of PN, entries 21–24, 2 × 10^−^^2^ mmol of NHC. ^b^ TOFs calculated at 1 h of reaction according to the surface amount of metal, in brackets TOF calculated for the hydrogenation reaction. Details in the calculation of the fraction of surface atoms are given in SI. ^c^ Determined by GC using an internal standard technique. * Other unidentified products.

## Data Availability

Not Applicable.

## Supplementary Materials

The following supporting information can be downloaded at https://www.mdpi.com/article/10.3390/nano12030328/s1, Figure S1: Time-concentration curve for furfural hydrogenation using Ru/PVP as catalyst; Table S1: Size before and after the hydrogenation of furfural in 1-PrOH using Ru/PVP nanocatalyst in situ modified; Figure S2: TEM image of Ru/PVP (scale bar 50 nm) together with the respective size histogram; Figure S3: TEM image of Ru/PVP after the hydrogenation of furfural in 1-PrOH at 20 bar of H_2_ pressure (scale bar 50 nm) together with the respective size histogram; Figure S4: TEM image of Ru/PVP after the hydrogenation of furfural in 1-PrOH at 20 bar of H_2_ pressure in the presence of HDA (scale bar 50 nm) together with the respective size histogram; Figure S5: TEM image of Ru/PVP after the hydrogenation of furfural in 1-PrOH at 20 bar of H_2_ pressure in the presence of TMP (scale bar 50 nm) together with the respective size histogram; Figure S6: TEM image of Ru/PVP after the hydrogenation of furfural in 1-PrOH at 20 bar of H_2_ pressure in the presence of TBA (scale bar 50 nm) together with the respective size histogram; Figure S7: TEM image of Ru/PVP after the hydrogenation of furfural in 1-PrOH at 20 bar of H_2_ pressure in the presence of PN (scale bar 50 nm) together with the respective size histogram; Figure S8: TEM image of Ru/PVP after the hydrogenation of furfural in 1-PrOH at 20 bar of H_2_ pressure in the presence of NHC (scale bar 50 nm) together with the respective size histogram.

## References

[1] Sun D., Sato S., Ueda W., Primo A., Garcia H., Corma A. (2016). Production of C4 and C5 alcohols from biomass-derived materials. Green Chem..

[2] Chen S., Wojcieszak R., Dumeignil F., Marceau E., Royer S. (2018). How Catalysts and Experimental Conditions Determine the Selective Hydroconversion of Furfural and 5-Hydroxymethylfurfural. Chem. Rev..

[3] Besson M., Gallezot P., Pinel C. (2014). Conversion of Biomass into Chemicals over Metal Catalysts. Chem. Rev..

[4] Mizugaki T., Kaneda K. (2019). Development of High Performance Heterogeneous Catalysts for Selective Cleavage of C−O and C−C Bonds of Biomass-Derived Oxygenates. Chem. Rec..

[5] Li X., Jia P., Wang T. (2016). Furfural: A Promising Platform Compound for Sustainable Production of C4 and C5 Chemicals. ACS Catal..

[6] Byun J., Han J. (2017). An integrated strategy for catalytic co-production of jet fuel range alkenes, tetrahydrofurfuryl alcohol, and 1,2-pentanediol from lignocellulosic biomass. Green Chem..

[7] Huang K., Brentzel Z.J., Barnett K.J., Dumesic J.A., Huber G., Maravelias C.T. (2017). Conversion of Furfural to 1,5-Pentanediol: Process Synthesis and Analysis. ACS Sustain. Chem. Eng..

[8] Smith H.A., Fuzek J.F. (1949). Catalytic Hydrogenation of Furan and Substituted Furans on Platinum. J. Am. Chem. Soc..

[9] Mizugaki T., Yamakawa T., Nagatsu Y., Maeno Z., Mitsudome T., Jitsukawa K., Kaneda K. (2014). Direct Transformation of Furfural to 1,2-Pentanediol Using a Hydrotalcite-Supported Platinum Nanoparticle Catalyst. ACS Sustain. Chem. Eng..

[10] Koch O., Koeckritz A., Kant M., Martin A., Schoening A., Armbruster U., Bartoszek M., Evert S., Lange B., Bienert R. (2012). Production of 1,2-pentanediol from Furfuryl Alcohol, Furfural and 1-hydroxy-2-Pentanone Using Heterogeneous Catalysts.

[11] Kaufmann W.E., Adams R. (1923). The use of platinum oxide as a catalyst in the reduction of organic compounds. IV. Reduction of furfural and its derivatives1. J. Am. Chem. Soc..

[12] Xu W., Wang H., Liu X., Ren J., Wang Y., Lu G. (2011). Direct catalytic conversion of furfural to 1,5-pentanediol by hydrogenolysis of the furan ring under mild conditions over Pt/Co2AlO4 catalyst. Chem. Commun..

[13] Ma R., Wu X.-P., Tong T., Shao Z.-J., Wang Y., Liu X., Xia Q., Gong X.-Q. (2016). The Critical Role of Water in the Ring Opening of Furfural Alcohol to 1,2-Pentanediol. ACS Catal..

[14] Tong T., Xia Q., Liu X., Wag Y. (2017). Direct hydrogenolysis of biomass-derived furans over Pt/CeO2 catalyst with high activity and stability. Catal. Commun..

[15] Zhang B., Zhu Y., Ding G., Zheng H., Li Y. (2012). Selective conversion of furfuryl alcohol to 1,2-pentanediol over a Ru/MnOx catalyst in aqueous phase. Green Chem..

[16] Goetz D., Lucas M., Claus P. (2016). C-O bond hydrogenolysis vs. C=C group hydrogenation of furfuryl alcohol: Towards sustainable synthesis of 1,2-pentanediol. React. Chem. Eng..

[17] Wang X., Weng Y., Zhao X., Xue X., Meng S., Wang Z., Zhang W., Duan P., Sun Q., Zhang Y. (2020). Selective hydrogenolysis and hydrogenation of furfuryl alcohol in the aqueous phase using Ru-Mn-based catalysts. Ind. Eng. Chem. Res..

[18] Cui K., Qian W., Shao Z., Zhao X., Gong H., Wei X., Wang J., Chen M., Cao X., Hou Z. (2021). Ru Nanoparticles on a Sulfonated Carbon Layer Coated SBA-15 for Catalytic Hydrogenation of Furfural into 1, 4-pentanediol. Catal. Lett..

[19] Yamaguchi A., Murakami Y., Imura T., Wakita K. (2021). Hydrogenolysis of Furfuryl Alcohol to 1,2-Pentanediol Over Supported Ruthenium Catalysts. ChemistryOpen.

[20] Koso S., Furikado I., Shimao A., Miyazawa T., Kunimori K., Tomishige K. (2009). Chemoselective hydrogenolysis of tetrahydrofurfuryl alcohol to 1,5-pentanediol. Chem. Commun..

[21] Nakagawa Y., Tomishige K. (2012). Production of 1,5-pentanediol from biomass via furfural and tetrahydrofurfuryl alcohol. Catal. Today.

[22] Tomishige K., Nakagawa Y., Tamura M., Fang Z., Smith R.L.J., Qi X. (2017). Production of diols from biomass. Production of Platform Chemicals from Sustainable Resources Biofuels and Biorefineries.

[23] Liu H., Huang Z., Zhao F., Cui F., Li X., Xia C., Chen J. (2016). Efficient hydrogenolysis of biomass-derived furfuryl alcohol to 1,2- and 1,5-pentanediols over a non-precious Cu–Mg3AlO4.5 bifunctional catalyst. Catal. Sci. Technol..

[24] Liu H., Huang Z., Kang H., Xia C., Chen J. (2016). Selective hydrogenolysis of biomass-derived furfuryl alcohol into 1,2- and 1,5-pentanediol over highly dispersed Cu-Al2O3 catalysts. Chin. J. Catal..

[25] Shao Y., Wang J., Du H., Sun K., Zhang Z., Zhang L., Li Q., Zhang S., Liu Q., Hu X. (2020). Importance of Magnesium in Cu-Based Catalysts for Selective Conversion of Biomass-Derived Furan Compounds to Diols. ACS Sustain. Chem. Eng..

[26] Yeh J.-Y., Matsagar B.M., Chen S.S., Sung H.-L., Tsang D.C., Li Y.-P., Wu K.C.-W. (2020). Synergistic effects of Pt-embedded, MIL-53-derived catalysts (Pt@Al_2_O_3_) and NaBH4 for water-mediated hydrogenolysis of biomass-derived furfural to 1,5-pentanediol at near-ambient temperature. J. Catal..

[27] Zhu Y., Zhao W., Zhang J., An Z., Ma X., Zhang Z., Jiang Y., Zheng L., Shu X., Song H. (2020). Selective activation of C-OH, C-O-C, or C=C in furfuryl alcohol by engineered Pt Sites supported on layered double oxides. ACS Catal..

[28] Axet M.R., Philippot K. (2020). Catalysis with Colloidal Ruthenium Nanoparticles. Chem. Rev..

[29] Cardona-Farreny M., Lecante P., Esvan J., Dinoi C., del Rosal I., Poteau R., Philippot K., Axet M.R. (2021). Bimetallic RuNi nanoparticles as catalysts for upgrading biomass: Metal dilution and solvent effects on selectivity shifts. Green Chem..

[30] Axet M.R., Conejero S., Gerber I.C. (2018). Ligand Effects on the Selective Hydrogenation of Nitrobenzene to Cyclohexylamine Using Ruthenium Nanoparticles as Catalysts. ACS Appl. Nano Mater..

[31] Min Y., Nasrallah H., Poinsot D., Lecante P., Tison Y., Martinez H., Roblin P., Falqui A., Poteau R., del Rosal I. (2020). 3D Ruthenium Nanoparticle Covalent Assemblies from Polymantane Ligands for Confined Catalysis. Chem. Mater..

[32] Luo Z., Min Y., Nechiyil D., Bacsa W., Tison Y., Martinez H., Lecante P., Gerber I.C., Serp P., Axet M.R. (2019). Chemoselective reduction of quinoline over Rh–C60 nanocatalysts. Catal. Sci. Technol..

[33] Leng F., Gerber I.C., Lecante P., Moldovan S., Girleanu M., Axet M.R., Serp P. (2016). Controlled and Chemoselective Hydrogenation of Nitrobenzene over Ru@C60 Catalysts. ACS Catal..

[34] Lu L., Zou S., Fang B. (2021). The Critical Impacts of Ligands on Heterogeneous Nanocatalysis: A Review. ACS Catal..

[35] Zhang S., Xia Z., Ni T., Zhang Z., Ma Y., Qu Y. (2018). Strong electronic metal-support interaction of Pt/CeO_2_ enables efficient and selective hydrogenation of quinolines at room temperature. J. Catal..

[36] Cao Z., Kim D., Hong D., Yu Y., Xu J., Lin S., Wen X., Nichols E., Jeong K., Reimer J.A. (2016). A Molecular Surface Functionalization Approach to Tuning Nanoparticle Electrocatalysts for Carbon Dioxide Reduction. J. Am. Chem. Soc..

[37] Chen G., Xu C., Huang X., Ye J., Gu L., Li G., Tang Z., Wu B., Yang H., Zhao Z. (2016). Interfacial electronic effects control the reaction selectivity of platinum catalysts. Nat. Mater..

[38] Lu G., Zhang P., Sun D., Wang L., Zhou K., Wang Z.-X., Guo G.-C. (2014). Gold catalyzed hydrogenations of small imines and nitriles: Enhanced reactivity of Au surface toward H2via collaboration with a Lewis base. Chem. Sci..

[39] Palazzolo A., Carenco S. (2021). Phosphines Modulating the Catalytic Silane Activation on Nickel–Cobalt Nanoparticles, Tentatively Attributed to Frustrated Lewis Pairs in a Colloidal Solution. Chem. Mater..

[40] Stephan D.W. (2021). Diverse Uses of the Reaction of Frustrated Lewis Pair (FLP) with Hydrogen. J. Am. Chem. Soc..

[41] Jafarpour L., Stevens E.D., Nolan S. (2000). A sterically demanding nucleophilic carbene: 1,3-bis(2,6-diisopropylphenyl)imidazol-2-ylidene). Thermochemistry and catalytic application in olefin metathesis. J. Organomet. Chem..

[42] Leng F., Gerber I.C., Axet M.R., Serp P. (2018). Selectivity shifts in hydrogenation of cinnamaldehyde on electron-deficient ruthenium nanoparticles. Comptes Rendus Chim..

[43] Cusinato L., Martínez-Prieto L.M., Chaudret B., del Rosal I., Poteau R. (2016). Theoretical characterization of the surface composition of ruthenium nanoparticles in equilibrium with syngas. Nanoscale.

[44] del Rosal I.A.P.R., Roucoux K.P.A.A. (2021). Sabatier principle and surface properties of small ruthenium nanoparticles and clusters: Case studies. Nanoparticles in Catalysis.

[45] Heuer-Jungemann A., Feliu N., Bakaimi I., Hamaly M., Alkilany A., Chakraborty I., Masood A., Casula M.F., Kostopoulou A., Oh E. (2019). The Role of Ligands in the Chemical Synthesis and Applications of Inorganic Nanoparticles. Chem. Rev..

[46] Pan C., Pelzer K., Philippot K., Chaudret B., Dassenoy F., Lecante P., Casanove M.-J. (2001). Ligand-Stabilized Ruthenium Nanoparticles: Synthesis, Organization, and Dynamics. J. Am. Chem. Soc..

[47] García-Antón J., Axet M.R., Jansat S., Philippot K., Chaudret B., Pery T., Buntkowsky G., Limbach H.-H. (2008). Reactions of Olefins with Ruthenium Hydride Nanoparticles: NMR Characterization, Hydride Titration, and Room-Temperature C-C Bond Activation. Angew. Chem. Int. Ed..

[48] Lara P., Rivada-Wheelaghan O., Conejero S., Poteau R., Philippot K., Chaudret B. (2011). Ruthenium Nanoparticles Stabilized by N-Heterocyclic Carbenes: Ligand Location and Influence on Reactivity. Angew. Chem. Int. Ed..

