# Supplementary material for: In Situ Ruthenium Catalyst Modification for the Conversion of Furfural to 1,2-Pentanediol
Source: Nanomaterials (Basel). 2022 Jan 20;12(3):328. doi: 10.3390/nano12030328 (PMC8840484; doi:10.3390/nano12030328)
Supplement: Supplementary file 1 [file nanomaterials-12-00328-s001.zip › nanomaterials-1494165-supplementary.pdf]

Supplementary Materials

In Situ Ruthenium Catalyst Modification for the Conversion of Furfural to 1,2-Pentanediol

Lauriane Bruna, Miquel Cardona-Farreny, Vincent Colliere, Karine Philippot and M. Rosa Axet *

CNRS, LCC (Laboratoire de Chimie de Coordination), Université de Toulouse, UPS, INPT,
205 route de Narbonne, CEDEX 04, 31077 Toulouse, France;
bruna.lauriane98@gmail.com (L.B.);
miquel.cardona@lcc-toulouse.fr (M.C.-F.); vincent.colliere@lcc-toulouse.fr (V.C.);
karine.philippot@lcc-toulouse.fr (K.P.)
* Correspondence: rosa.axet@lcc-toulouse.fr

Fraction of surface atoms

The number of Ru atoms in *hcp* cell (N) is 6. Ru atom radius (R_{Ru}) is 0.214 nm. The volume of Ru cell is 0.0817 nm^3. R_{NP} represents the radius of a NP, determined using TEM images.
The volume of all Ru atoms on the shell of NP: $V_{shell} = V_{total} - V_{core} = 4/3\pi R_{NP}^3 - 4/3\pi(R_{NP} - R_{Ru})^3$, V_{total} meaning the volume of one Ru nanoparticle, V_{core} presenting the volume of NP excluded the one outer layer of atoms.
The numbers of metal atoms on the shell $N_{shell} = N*V_{shell} /0.0817$.
The number of total Ru atoms $N_{total} = N*V_{total}/0.0817$.
The percentage of Ru atoms on the surface of NP = N_{shell}/N_{total} *100%.
Taking into consideration these calculations for a 1.6 nm Ru NP the % of Ru surface atoms is 61.

Arblaster, J. W., Crystallographic properties of ruthenium. *Platinum Met. Rev.* **2013,** *57*, 127-136.

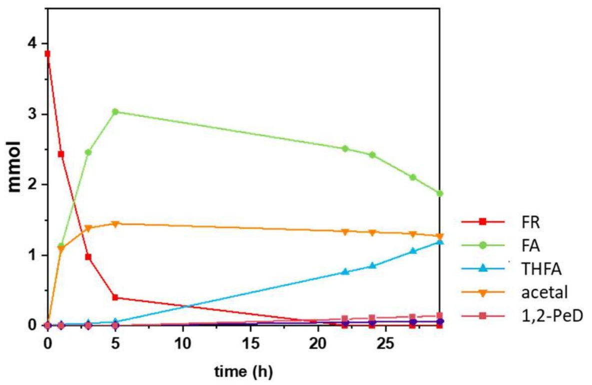

Figure S1. Time-concentration curve for furfural hydrogenation using Ru/PVP as catalyst. Furfural (red curve), furfuryl alcohol (green curve), tetrahydrofurfuryl alcohol (blue curve), acetal (orange curve), 1,2-pentanediol (pink curve). Reaction conditions: 2 × 10^{-2} mmol of Ru, 4 mmol of FA, 0.5 mmol of dodecane (internal standard), 20 bar of H_2, 125°C, 15 mL of 1-propanol. Quantities of products and reagents were determined by GC using an internal standard technique.

M. Cardona-Farreny, P. Lecante, J. Esvan, C. Dinoi, I. Del Rosal, R. Poteau, K. Philippot, M.R. Axet, Bimetallic RuNi nanoparticles as catalysts for upgrading biomass: metal dilution and solvent effects on selectivity shifts, Green Chem., 23 (2021) 8480-8500.

Table S1. Size before and after the hydrogenation of furfural in 1-PrOH using Ru/PVP nanocatalyst in situ modified.

Entry	Catalyst	Size before reaction (nm)[a]	Size after reaction (nm)[a]	Leaching (%)[b]
1	Ru/PVP		1.6 ± 0.3	0.8
2	Ru/PVP + HDA		1.5 ± 0.3	1.6
3	Ru/PVP + TMP	1.6 ± 0.3	1.6 ± 0.4	3.0
4	Ru/PVP + TBA		1.4 ± 0.4	1.3
5	Ru/PVP + PN		1.6 ± 0.4	0.1
6	Ru/PVP + NHC		1.7 ± 0.4	2.9

[a]Mean values of nanoparticle size determined from TEM images by considering at least 200 particles.
[b]Determined by ICP analysis.

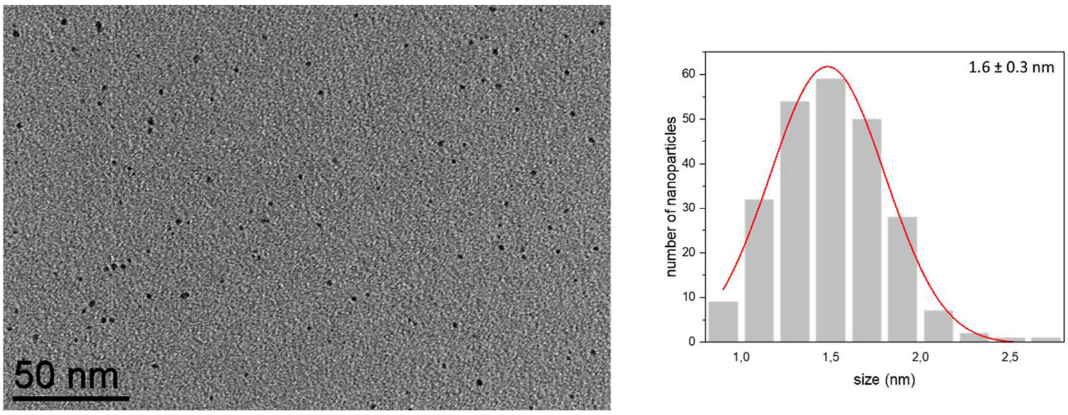

Figure S2. TEM image of Ru/PVP (scale bar 50 nm) together with the respective size histogram.

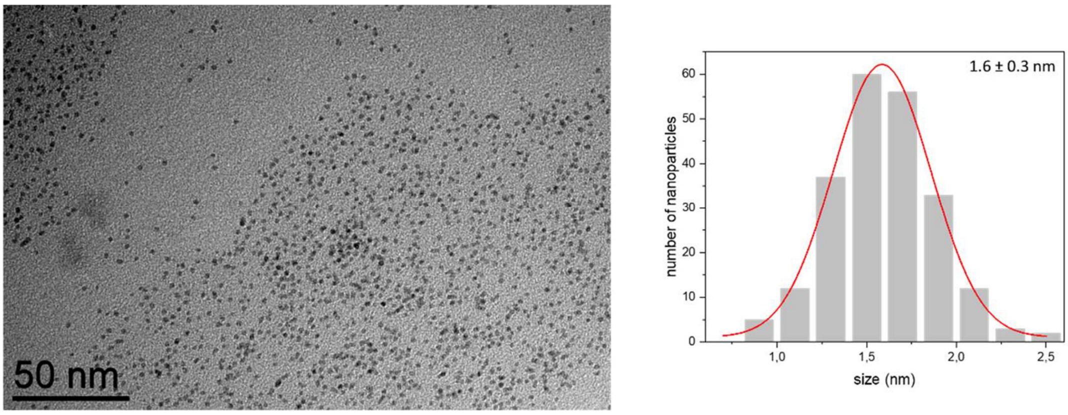

Figure S3. TEM image of Ru/PVP after the hydrogenation of furfural in 1-PrOH at 20 bar of H_2 pressure (scale bar 50 nm) together with the respective size histogram.

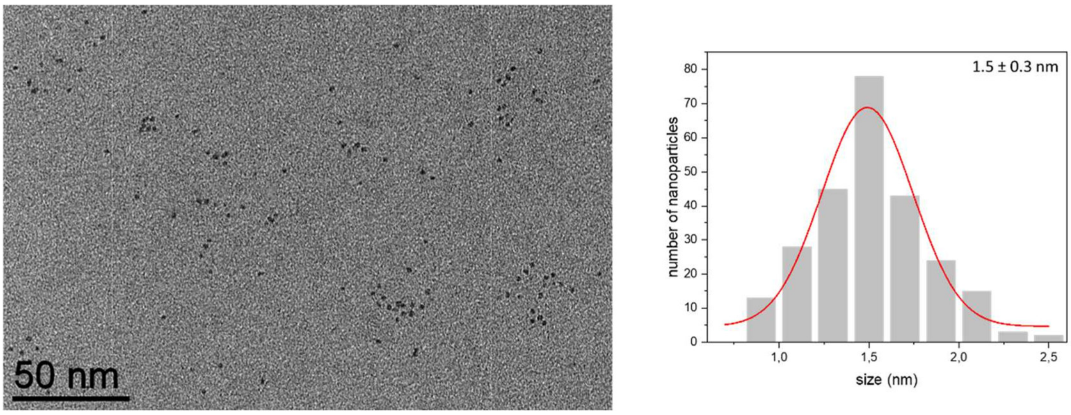

Figure S4. TEM image of Ru/PVP after the hydrogenation of furfural in 1-PrOH at 20 bar of H_2 pressure in the presence of HDA (scale bar 50 nm) together with the respective size histogram.

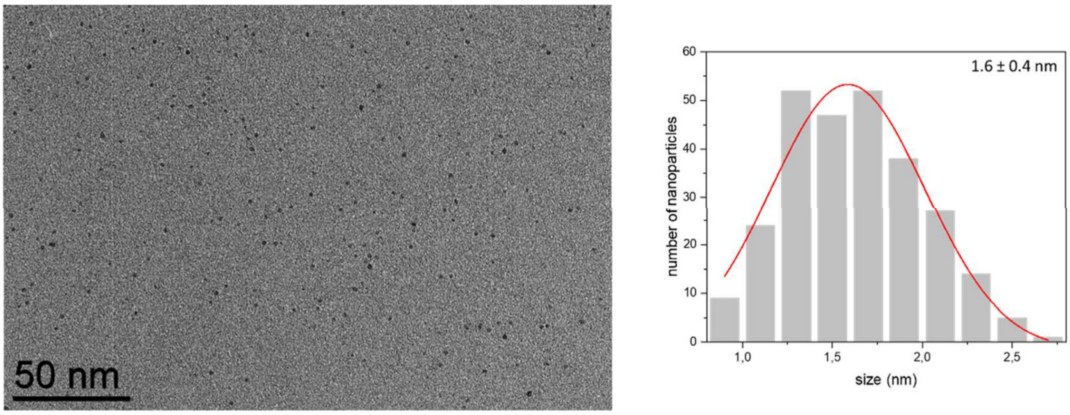

Figure S5. TEM image of Ru/PVP after the hydrogenation of furfural in 1-PrOH at 20 bar of H_2 pressure in the presence of TMP (scale bar 50 nm) together with the respective size histogram.

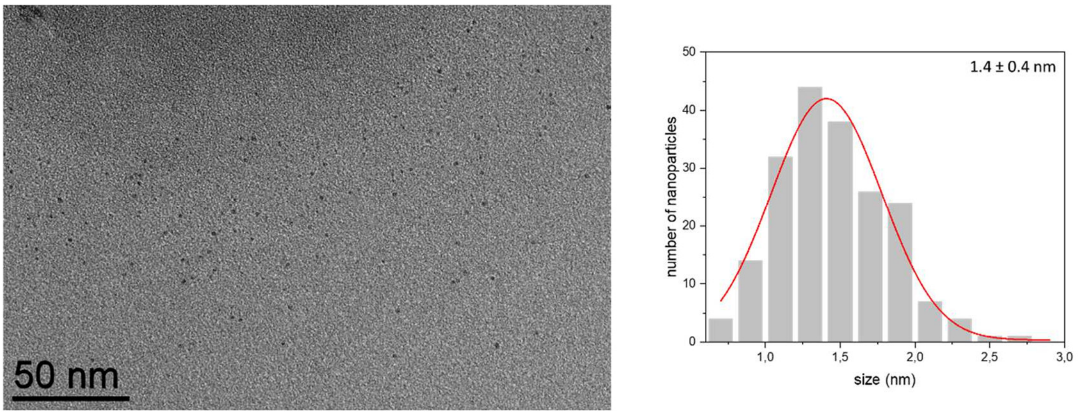

Figure S6. TEM image of Ru/PVP after the hydrogenation of furfural in 1-PrOH at 20 bar of H_2 pressure in the presence of TBA (scale bar 50 nm) together with the respective size histogram.

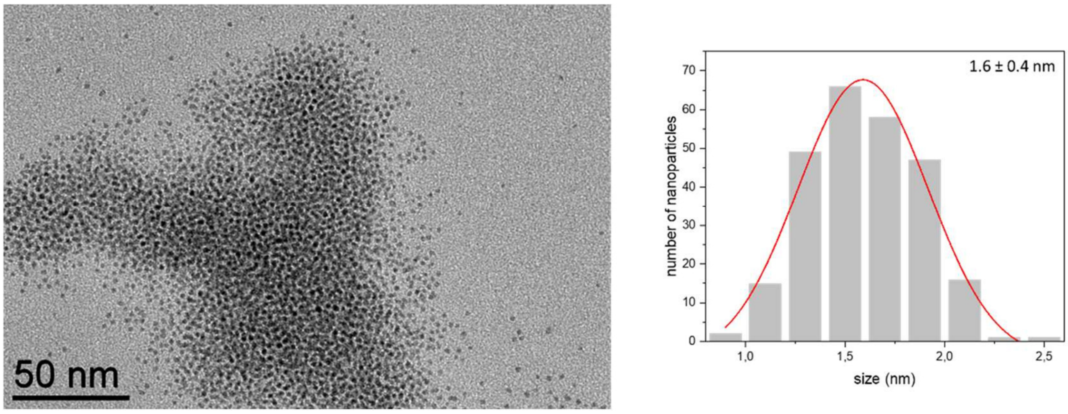

Figure S7. TEM image of Ru/PVP after the hydrogenation of furfural in 1-PrOH at 20 bar of H_2 pressure in the presence of PN (scale bar 50 nm) together with the respective size histogram.

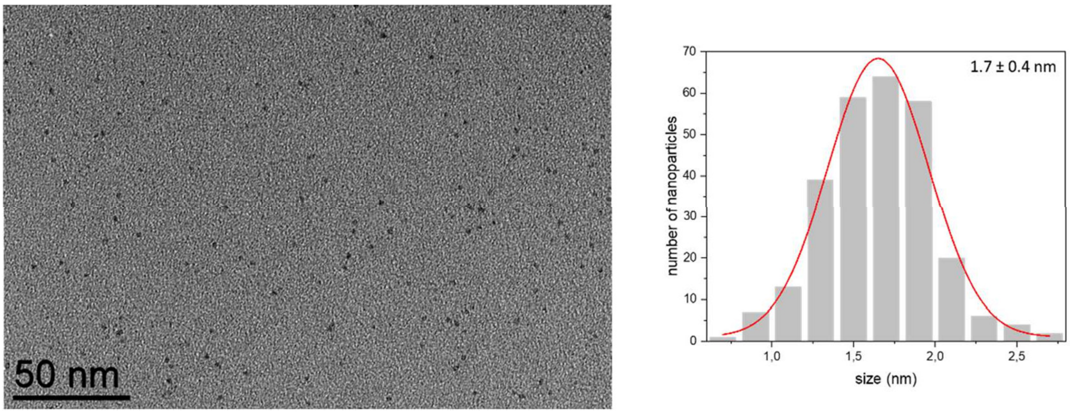

Figure S8. TEM image of Ru/PVP after the hydrogenation of furfural in 1-PrOH at 20 bar of H_2 pressure in the presence of NHC (scale bar 50 nm) together with the respective size histogram.
